# The quality of telepsychiatry in terms of accessibility, appropriateness, effectiveness, and safety among psychiatric patients in King Abdulaziz Medical City: An observational cross-sectional analytical study

**DOI:** 10.1097/MD.0000000000030499

**Published:** 2022-09-16

**Authors:** Gada Wafia, Ola Bahadur, Salman Thabet, Moayyad Alsalem, Muhammad Anwar Khan, Majed A. Alharbi, Ahmad Alsaleh

**Affiliations:** a College of Medicine, King Saud bin Abdulaziz University for Health Sciences, Jeddah, Kingdom of Saudi Arabia; b Psychiatry Section, Department of Medicine, King Abdulaziz Medical City, Ministry of the National Guard- Health Affairs, Jeddah, Kingdom of Saudi Arabia; c King Abdullah International Medical Research Center, Jeddah, Kingdom of Saudi Arabia; d Ministry of the National Guard- Health Affairs Riyadh. Kingdom of Saudi Arabia; e Department of Adult Mental Health, King Abdulaziz Medical City, Jeddah, Kingdom of Saudi Arabia.

**Keywords:** CSQ-18, mental health, patient’s satisfaction, psychiatry, survey, telepsychiatry

## Abstract

There has been an increasing demand for psychiatric care in recent decades, and “telepsychiatry” was developed to meet these demands. It is a type of telemedicine in which they provide many medical services virtually, such as therapy, counseling, and medication management. Telepsychiatry has numerous advantages, including lower costs, reduced stigma, and improved continuity of care. To the best of our knowledge, no previous studies in the western region of Saudi Arabia addressed patients satisfaction with telepsychiatry. This cross-sectional study aims to assess patient satisfaction in telepsychiatry in terms of accessibility and timeliness, appropriateness, effectiveness, and safety, and to see whether patient satisfaction affects their decision to use the service again in the future. A cross-sectional study was conducted using a prestructured survey on the basis of the Client Satisfaction Questionnaire-18, which is a validated questionnaire used to assess patients’ satisfaction with the services provided to them. From January 2021 to July 2021, all male and female psychiatric patients over the age of 18 years who had psychiatric virtual appointments were included in this study. This study included 182 patients, of whom 106 were female. Patients were generally satisfied with the telepsychiatry services; 56.6%, 81.9%, 86.8%, and 91.2% of the participants were satisfied with the access and timeliness, appropriateness, effectiveness, and safety, respectively, and a total of 58.3% either strongly agree or agree of the overall satisfaction level. Depression and anxiety disorders were the most common psychiatric diseases. The statistical analysis revealed no significant relationships between patients’ satisfaction and demographic characteristics. Telepsychiatry has been evaluated to meet the growing demand for psychiatric care; it also has significant advantages. Patients had an overall positive satisfaction level toward telepsychiatry service, and so the results of this study support the continuity of using telepsychiatry in the future. Further research area could include a comparison between patients’ and providers’ satisfaction levels with telepsychiatry.

## 1. Introduction

The highly contagious coronavirus disease 2019 (COVID-19) first appeared in China at the end of 2019, and it quickly became a pandemic at the start of 2020.^[1]^ Consequently, the population was forced to live in isolation, and health care providers had to become creative to continue providing high-quality patient care.^[1]^ The COVID-19 pandemic has had a significant impact on telepsychiatry, which is a type of telemedicine in which they provide many medical services virtually, such as therapy, counseling, and medication management.^[2]^ Telepsychiatry has numerous advantages, including lower costs, reduced stigma, and improved continuity of care.^[2]^

For health care employees, patient satisfaction feedback offers priceless information that is used to improve the quality of services.^[3,4]^ A study on the level of satisfaction for psychiatric patients was conducted in the United States, with 22 patients participating for 12 weeks.^[3]^ The assessment was carried at 3 separate sessions using the Client Satisfaction Questionnaire-8 (CSQ-8), which is one of the standardized satisfaction measures that have been used across mental health services; they reported high levels of satisfaction.^[3,4]^ In a cross-sectional study that was conducted in Australia (n = 1378), the results concluded that 86% of patients were satisfied.^[4]^

Another study that used telephone surveys included 156 patients over a 6-month period, with 121 patients (78%) completing the questionnaire.^[5]^ The study found that 80% of patients were satisfied, and 63% said they would use telemedicine again. More than half of the participants believed that telemedicine had a positive impact in terms of medical care improvement.^[5]^ Also, in a 2-year study conducted across 17 medical centers, they used a 21-question survey based on the Client Satisfaction Questionnaire-18 (CSQ-18), which assesses factors such as access and timeliness, appropriateness, effectiveness, and safety, by using the 5-point Likert scale.^[6]^ They had 274 patients, who had an overall satisfaction of 91%.^[6]^

In Australia, telemedicine is used in many mental health centers when dealing with patients from rural areas.^[7]^ A prospective study comparing a group of patients who had face-to-face appointments (n = 92) and a group who had online appointments (n = 32) for 2 years found that there was no significant difference between the 2 groups in effectiveness.^[7]^ This suggests that telepsychiatry has a significant role in the delivery of mental health services.

In conclusion, telepsychiatry has been evaluated to meet the growing demand for psychiatric care. It also has significant advantages, such as the ability to reach more people including those living in rural communities. The utility of telepsychiatry is relevant now more than ever because of the COVID-19 pandemic because it could mean a change in clinic practice could be considered, and using a validated client satisfaction questionnaire, which is used in many studies worldwide, had provided essential information for advancement in the medical health care field. Thus, this study aims to measure the level of satisfaction in psychiatric patients, who had virtual appointments during the COVID-19 crisis to Investigate whether satisfaction affects patients’ well-being.

## 2. Materials and Methods

A cross-sectional, observational study from January to July of 2021 was conducted to measure the level of satisfaction in psychiatric patients who had virtual appointments during the COVID-19 pandemic and assess their satisfaction in terms of access and timeliness, appropriateness, effectiveness, and safety at the National Guard Hospital in King Abdelaziz Medical City, Jeddah, a tertiary health care center. The hospital presents the western region population with different medical services. Ethical approval was obtained from the Institutional Review Board at King Abdullah International Medical Research Center, project number NRJ21J/273/11. For quantitative data collection, a structured telephone interview with a questioner will be used. From January 2021 to July 2021, we included all male and female psychiatric patients over the age of 18 years, who had psychiatric virtual appointments. Patients who never had an on-site appointment, those who had no contact information in the databases, those who refused to answer the survey, those who provided the incorrect telephone number, or those who did not respond after 2 attempts were excluded.

Previous studies on this subject showed a response rate range of 80% to 91% and by taking the average, it would be 85.5%; with that, we used the Raosoft sample size calculator to calculate a sample with a margin error of 5%, the confidence level of 95%, and estimated population size of 400 patients per month. For a 6-month period, the calculated sample size was 177 patients. We chose our sample from the above group using a nonprobability convenient sampling technique, with subjects chosen based on their accessibility and criteria requirements.

After Institutional Review Board approval was obtained, the study was conducted by medical students from the National Guard Hospital, Jeddah, Saudi Arabia. After reviewing the literature to identify a validated questionnaire, the CSQ-18 was chosen, the validity and reliability of which were tested and verified in literature by Serhal et al.^[6]^ The reliability was calculated using the method outlined by RayKov, which can be interpreted similarly as “the coefficient alpha: the closer to 1 its value is, the higher the reliability, with values >0.7 being considered acceptably reliable,”^[8,9]^ and permission from creators was taken by official email. The CSQ-18 questionnaire is an 18-item scale that is divided into assessing 4 factors, namely “access and timeliness” with lower but still acceptable reliability of 0.72, “appropriateness,” “effectiveness,” and “safety” with good reliability of 0.81, 0.83, and 0.86, respectively. The participants were asked to rate these items on a Likert scale of 1 (strongly disagree) to 5 (strongly agree), where the maximum score will be 90 points. A total score equal to 18 or less was considered “very Low,” from 19 to 36 was “low,” from 37 to 54 was “moderate,” from 55 to 72 was considered “high,” and from 73 to 90 was considered “very high.”

Following the selection of participants who met the inclusion and exclusion criteria, their voluntary informed consent was obtained at the start of the interview. They were then interviewed over the phone. The “Bestcare” database, which is the hospital electronic database, was used to obtain contact and sociodemographic information for all patients. The interviewers were in charge of making phone calls and recording data for the analysis. Before administering the survey, the questionnaire was translated into Arabic and validated by giving the Arabic version to an associate who translated it into English, which was then compared with the original questionnaire.

Our methodology for making the calls was that if the first phone call went unanswered, another call would be made at a different time of day, where the following occurrences were marked as a “NO ANSWER”: no answer for all of the 2 calls, the dialed number did not match the patient, and there was no contact information recorded in the databases.

Only data relevant to this study as outlined in this protocol were collected by the research team. All the information collected during the research project remained confidential to the extent required and provided by law. Patient data were anonymized. No code linking patient identifiers to patient data was kept, and it will not be possible to identify participants. Collected data were documented using the hospital’s computers that are secured with a password with only access to authorized personnel. Lastly, their data were transferred onto a data collection sheet. The variables that were measured were demographic data that included age, gender, marital status, education degree, place of residence (rural or in the city), psychiatric admissions in the last year, and information regarding the teleconsultations (diagnosis and number of sessions).

Microsoft Office Excel software was used for data entry, and the analysis was done by using IBM Statistical Package for Social Sciences (SPSS) version 24 program (IBM; SPSS Statistics for Windows. Version 24.0. Armonk, NY: IBM Crop; 2017). Qualitative and demographic data will be described as mean and standard deviation or median and range as appropriate and would be represented as frequencies and percentages. For comparative analysis, chi-square and Fisher exact test will be used, with a *P* value of <.05 to indicate the statistical significance.

## 3. Results

For this study, which had a response rate of 90.54%, 182 patients were included, of whom 58.2% were female. The mean age of the participants was 43 years old, with a standard deviation of 14.4. Out of those, 61.5% were married, 18.7% were single, and 12.6% were divorced. The majority lived in Jeddah 56.9%. The educational level of the subjects was divided into “less than high school level” 44%, “high school degree” 31.9%, “college undergraduate” 10.4%, and “college degree” 13.7% (Table 1).

**Table 1:** Demographic data and telepsychiatry exposure.

	Participants, n = 182 (100%)
Gender	
Female	106 (58.2%)
Age (mean)	43.7 yr
Age (standard deviation)	14.4 yr
Residing city	
Jeddah	120 (56.9%)
Makkah	29 (15.9%)
Taif	15 (8.2%)
Other	19 (9.9%)
Marital status	
Married	112 (61.5%)
Single	34 (18.7%)
Divorced	23 (12.6%)
Widowed	13 (7.1%)
Educational level	
Less than high school	80 (44%)
High school	58 (31.9%)
College undergraduate	19 (10.4%)
College degree	25 (13.7%)
Psychiatric diagnosis	
Major depressive disorder	56 (30.8%)
Generalized anxiety disorder	39 (21.4%)
Bipolar disorder	21 (11.5%)
Mixed anxiety and depression	17 (9.3%)
Schizophrenia	13 (7.1%)
Other	36 (19.9%)
Telepsychiatry exposure	
1	42 (23.1%)
2–4	84 (46.2%)
5	56 (30.8%)
Psychiatric admissions (2020–2021)	58 (31.9%)

n = sample size.

The patients’ psychiatric diagnoses recorded were major depressive disorder 30.8%, generalized anxiety disorder 21.4%, bipolar disorder 11.5%, mixed anxiety and depression 9.3%, and schizophrenia 7.1%, and the rest were classified under other 19.9% (Table 1).

The patients’ telepsychiatry exposure was divided into 3 categories: patients who had 1 telepsychiatry session 23.1%, those who had 2 to 4 sessions 46.2%, and those who had 5 or more sessions, 30.8%. Also, psychiatric admission between the years 2020 and 2021 was documented; 31.9% of the patients had been admitted to a psychiatric hospital (Table 1).

Generally, patients reported a positive response with telepsychiatry appointments; 30.9% of the patients strongly agree with the overall satisfaction as well as 27.4% of the patients agree with the overall satisfaction, which makes 58.3% of total patients either satisfied or strongly satisfied overall. Whereas 15.9% of the participant reported a neutral response. The complete results for all survey questions are mentioned in Table 2.

**Table 2:** Survey questions.

	Strongly disagree	Disagree	Neutral	Agree	Strongly agree
Q 1: “I am satisfied with the length of time I had to wait between my referral and the telepsychiatry appointment.”	35 (19.2%)	42 (23.1%)	18 (9.9%)	44 (24.2%)	43 (23.6%)
Q 2: “It was easy to book my telepsychiatry appointment.”	15 (8.2%)	29 (15.9%)	73 (40.1%)	35 (19.2%)	30 (16.5%)
Q 3: “I was able to get an appointment through Telepsychiatry sooner than an in-person psychiatry appointment.”	19 (10.4%)	31 (17%)	72 (39.6%)	28 (15.4%)	32 (17.6%)
Q 4: “The physical location of my telepsychiatry appointment was convenient for me to get to.”	14 (7.7%)	30 (16.5%)	31 (17%)	42 (23.1%)	65 (35.7%)
Q 5: “I believe Telepsychiatry is just as effective as an in-person psychiatry appointment.”	20 (11%)	29 (15.9%)	26 (14.3%)	42 (23.1)	65 (35.7%)
Q 6: “The psychiatrist understood my concerns during the virtual Mental health appointment”.	9 (4.9%)	16 (8.8%)	26 (14.3%)	38 (20.9%)	93 (51.1%)
Q 7: “The psychiatrist involved me in decisions about my treatment plan.”	5 (2.7%)	21 (11.5%)	17 (9.3%)	34 (18.7%)	105 (57.7%)
Q 8: “I am confident that I will be able to follow the psychiatrist’s recommendations.”	9 (4.9%)	19 (10.4%)	19 (10.4%)	32 (17.6%)	103 (56.6%)
Q 9: “During my telepsychiatry appointment, I was able to hear the psychiatrist clearly.”	3 (1.6%)	16 (8.8%)	12 (6.6%)	22 (12.1%)	129 (70.9%)
Q 10: “I am confident that the psychiatrist and my health care providers are working as a team.”	4 (2.2%)	19 (10.4%)	17 (9.3%)	31 (17%)	111 (61%)
Q 11: “I feel that there was an adequate amount of time allotted for the telepsychiatry appointment.”	4 (2.2%)	20 (11%)	10 (5.5%)	36 (19.8%)	112 (61.5%)
Q 12: “I felt comfortable during my telepsychiatry appointment.”	15 (8.2%)	28 (15.4%)	25 (13.7%)	42 (23.1)	72 (39.6)
Q 13: “I felt that my confidentiality was protected throughout my telepsychiatry appointment.”	1 (0.5%)	13 (7.1%)	27 (14.8%)	34 (18.7%)	107 (58.8%)
Q 14: “The psychiatrist treated me with courtesy and respect.”	2 (1.1%)	16 (8.8%)	11 (6%)	29 (15.9%)	124 (68.1%)
Q 15: “The psychiatrist explained my diagnosis in a way that I could understand.”	2 (1.1%)	20 (11%)	15 (8.2%)	33 (18.1%)	112 (61.5%)
Q 16: “The psychiatrist explained the benefits and risks of any medications he/she recommended.”	8 (4.4%)	36 (19.8%)	37 (20.3%)	27 (14.8%)	74 (40.7%)
Q 17: “I understand what to do if I have a mental health emergency following this appointment.”	24 (13.2%)	35 (19.2%)	31 (17%)	33 (18.1%)	59 (32.4%)
Q 18: “Overall, I’m satisfied with my virtual mental health appointment.”	16 (8.8%)	31 (17%)	29 (15.9%)	52 (27.4%)	54 (30.9%)

Overall, the client satisfaction survey demonstrated high rating from patients across the 4 domains. For the access and timeliness category, 56.6% of participants were highly satisfied. For safety, 91.2% were highly satisfied with telepsychiatry. Regarding to effectiveness and appropriateness, participants showed a very high level of satisfaction, 86.8% and 81.9%, respectively. Thus, the highest overall satisfaction was for effectiveness. The total factor score was calculated for each factor by summing the scores for each item. We observed that for all factors, total scores were concentrated on the higher end of the scale, as shown in Table 3.

**Table 3:** Level of satisfaction for each category.

	n = 182 (100%)				
	Very low	Low	Moderate	High	Very high
Factor 1: Access and timeliness	0 (0.0%)	15 (8.2%)	64 (35.2%)	77 (42.3%)	26 (14.3%)
Q 1: “I am satisfied with the length of time I had to wait between my referral and the Telepsychiatry appointment.”					
Q 2: “It was easy to book my Telepsychiatry appointment.”					
Q 3: “I was able to get an appointment through Telepsychiatry sooner than an in-person psychiatry appointment.”					
Q 4: “The physical location of my Telepsychiatry appointment was convenient for me to get to.”					
Factor 2: Appropriateness	1 (0.5%)	7 (3.8%)	25 (13.7%)	58 (31.9%)	91 (50.0%)
Q 5: “I believe Telepsychiatry is just as effective as an in-person psychiatry appointment.”					
Q 6: “The psychiatrist understood my concerns during the virtual Mental health appointment.”					
Q 7: “The psychiatrist involved me in decisions about my treatment plan.”					
Q 8: “I am confident that I will be able to follow the psychiatrist’s recommendations.”					
Factor 3: Effectiveness	0 (0.0%)	3 (1.6%)	21 (11.5%)	41 (22.5%)	117 (64.3%)
Q 9: “During my Telepsychiatry appointment, I was able to hear the psychiatrist clearly.”					
Q 10: “I am confident that the psychiatrist and my health care providers are working as a team.”					
Q 11: “I feel that there was an adequate amount of time allotted for the telepsychiatry appointment.”					
Factor 4: Safety	0 (0.0%)	1 (0.5%)	15 (8.2%)	85 (46.7%)	81 (44.5%)
Q 12: “I felt comfortable during my Telepsychiatry appointment.”					
Q 13: “I felt that my confidentiality was protected throughout my Telepsychiatry appointment.”					
Q 14: “The psychiatrist treated me with courtesy and respect.”					
Q 15: “The psychiatrist explained my diagnosis in a way that I could understand.”					
Q 16: “The psychiatrist explained the benefits and risks of any medications he/she recommended.”					
Q 17: “I understand what to do if I have a mental health emergency following this appointment.”					

n = sample size.

The statistical analysis revealed no significant relationships between patients’ satisfaction and demographic characteristics (Table 4).

**Table 4:** Level of total satisfaction and significance levels.

	Overall, I’m satisfied with my virtual mental health appointment										
	Strongly disagree		Disagree		Neutral		Agree		Strongly agree		*P*
	n = 16	%	n = 31	%	n = 29	%	n = 53	%	n = 53	%	
Diagnosis											.780
MDD	6	10.7	6	10.7	7	12.5	18	32.1	19	33.9	
GAD	3	7.7	10	25.6	4	10.3	10	25.6	12	30.8	
Mixed anxiety and depression	2	11.8	2	11.8	4	23.5	5	29.4	4	23.5	
Bipolar disorder	1	4.8	5	23.8	3	14.3	7	33.3	5	23.8	
Schizophrenia	2	15.4	1	7.7	2	15.4	2	15.4	6	46.2	
Other	2	5.6	7	19.4	9	25.0	11	30.6	7	19.4	
Residing city											**.378**
Jeddah	10	8.3	19	15.8	15	12.5	36	30.0	40	33.3	
Makkah	2	6.9	7	24.1	5	17.2	10	34.5	5	17.2	
Taif	3	20.0	2	13.3	3	20.0	2	13.3	5	33.3	
other	1	5.6	3	16.7	6	33.3	5	27.8	3	16.7	
Educational degree											**.536**
Less than high school	10	12.5	11	13.8	13	16.3	20	25.0	26	32.5	
High school graduate	4	6.9	12	20.7	8	13.8	15	25.9	19	32.8	
College undergraduate	1	5.3	4	21.1	3	15.8	6	31.6	5	26.3	
College postgraduate	1	4.0	4	16.0	5	20.0	12	48.0	3	12.0	
Marital Status											**.108**
single	4	11.8	3	8.8	4	11.8	15	44.1	8	23.5	
Married	9	8.0	16	14.3	20	17.9	29	25.9	38	33.9	
Divorced	3	13.0	9	39.1	3	13.0	5	21.7	3	13.0	
Widowed	0	0.0	3	23.1	2	15.4	4	30.8	4	30.8	
Telepsychiatry exposure:											**.200**
1	1	2.4	6	14.3	6	14.3	15	35.7	14	33.3	
2–4	8	9.5	18	21.4	10	11.9	27	32.1	21	25.0	
≥5	7	12.5	7	12.5	13	23.2	11	19.6	18	32.1	

Fisher exact test, if *P* value is <.05 then there will be statistical significance.

GAD = generalized anxiety disorder, MDD = major depressive disorder, *P* = probability value.

## 4. Discussion

This study used a validated and reliable questionnaire to assess patients’ overall satisfaction and experience with telepsychiatry across 4 domains: access and timeliness, appropriateness, effectiveness, and safety. This survey has universal importance in telepsychiatry as well as the other areas of telemedicine.^[6]^ In this study, the most prevalent psychiatric diseases were depression and anxiety disorders, 30.8% and 21.4%, respectively. It is following what is reported worldwide, where the mental illnesses with the highest prevalence in the general population are anxiety and mood disorders.^[10]^ A descriptive, cross-sectional observational study that was conducted in Colombia for presenting the level of satisfaction of 111 patients who participated in telepsychiatry found that the most prevalent diseases were depression and anxiety disorders, among which was panic disorder.^[11]^ In Australia, cross-sectional research was collected from across 14 Australian long-term alcohol and other drug treatment facilities (n = 1378); the most prevalent psychiatric disorders were depressive disorders at 38.5% and anxiety disorders at 14.9%.^[4]^

Previous research has found that telepsychiatry services are well liked by patients.^[4,7,12–15]^ Patients’ satisfaction was high in all 4 domains examined in this study, with the highest score recorded for effectiveness, followed by appropriateness, safety, and finally access and timeliness. This is consistent with the findings of a study conducted in Canada on 274 patients who received consultations through the Centre for Addiction and Mental Health’s TeleMental Health Program.^[6]^

The overall satisfaction level with telepsychiatry in this study was 58.3% of patients, which is consistent with other studies’ results. For example, a study in Canada that included 110 patients to assess their satisfaction and accessibility level reported that almost all patients were satisfied with the telepsychiatry session overall (96.3%). Also, patients largely agreed that telepsychiatry afforded them better access to care compared with face-to-face appointments.^[12]^ In 2018, a study done in Australia had 1378 participants from different healthcare facilities. Data from 1378 subjects were collected and the overall satisfaction level was 86%.^[4]^ In 2020, a Canadian study, which included 274 subjects, has concluded that 58% of the patients strongly agree with the overall satisfaction question.^[7]^ Lastly, in Saudi Arabia, a study was conducted on 141 patients, and they reported that 94.3% of the patients had a sense of overall satisfaction.^[16]^

In a systemic review for 44 articles that reported patient satisfaction. It should be noted that over the past 7 years in the included literature, the factors listed the most often were effectiveness (20%), accessibility (9%), low cost (8%), and decreased travel time (7%).^[13]^

The statistical analysis in this study revealed no significant relationship between patient satisfaction and any of the demographic characteristics mentioned. This is supported by the previously mentioned Australian study, which discovered no relationship between patient characteristics (age and gender) and satisfaction,^[4]^ as well as no significant relationship between satisfaction and educational level.^[15]^ Various reactions to the introduction of a new modality of care were mentioned in the literature. One study found significant resistance to change, whereas others reported an embrace of the change. Generally, older patients are resistant to change, but recent studies have found that telepsychiatry is becoming more popular.^[13]^

Finally, one of the limitations of this study is that most of the sample size was from an urban area, so it does not include patients from rural areas. Moreover, because this is a single-center study, the findings may be limited and should be more generalized. Future research could include psychiatrists’ perspectives on patient and provider satisfaction levels, as well as assessing and comparing them.

## 5. Conclusion

This research shows that patients in King Abdulaziz Medical City, Jeddah expressed high levels of satisfaction with the telepsychiatric consultation they received. Patients were satisfied with telepsychiatry in all 4 domains, with the highest score for effectiveness, followed by appropriateness, then safety, and finally access and timeliness. The vast majority of those who participated expressed an interest in using it in the future, and so the results of this study support the continuity of using telepsychiatry in the future. Depressive and anxiety disorders are the most common mental disorders. Further research is needed to include psychiatrists’ perceptions and a comparison between patients’ and providers’ satisfaction levels about telepsychiatry.

## Acknowledgments

The authors would like to thank Enago (www.enago.com) for the English language review.

## References

[1] Sullivan AB Kane A Roth AJ Davis BE Drerup ML Heinberg LJ. The COVID-19 crisis: a mental health perspective and response using telemedicine. J Patient Exp. 2020;7:295–301. 32821785 10.1177/2374373520922747 PMC7410128

[2] El Hayek S Nofal M Abdelrahman D. Telepsychiatry in the Arab world: a viewpoint before and during COVID-19. Neuropsychiatr Dis Treat. 2020;16:2805–15. 33239877 10.2147/NDT.S277224 PMC7682595

[3] Lee EB Haeger JA Levin ME Ong CW Twohig MP. Telepsychotherapy for trichotillomania: a randomized controlled trial of ACT enhanced behavior therapy. J Obsessive Compuls Relat Disord. 2018;18:106–15.

[4] Kelly PJ Kyngdon F Ingram I Deane FP Baker AL Osborne BA. The client satisfaction questionnaire-8: psychometric properties in a cross-sectional survey of people attending residential substance abuse treatment. Drug Alcohol Rev. 2018;37:79–86. 28480521 10.1111/dar.12522

[5] López C Valenzuela JI Calderón JE Velasco AF Fajardo R. A telephone survey of patient satisfaction with realtime telemedicine in a rural community in Colombia. J Telemed Telecare. 2011;17:83–7. 21139016 10.1258/jtt.2010.100611

[6] Serhal E Kirvan A Sanches M Crawford A. Client satisfaction and experience with telepsychiatry: development and validation of a survey using clinical quality domains. J Med Internet Res. 2020;22:e19198. 32755896 10.2196/19198 PMC7556368

[7] Kennedy C Yellowlees P. The effectiveness of telepsychiatry measured using the health of the nation outcome scale and the mental health inventory. J Telemed Telecare. 2003;9:12–6. 12641887 10.1258/135763303321159639

[8] Raykov T. Alpha if item deleted: a note on loss of criterion validity in scale development if maximizing coefficient alpha. Br J Math Stat Psychol. 2008;61:275–85. 17535482 10.1348/000711007X188520

[9] Cortina JM. What is coefficient alpha? An examination of theory and applications. J Appl Psychol. 1993;78:98.

[10] World Health Organization. Mental health: evidence and research team mental health in emergencies: mental and social aspects of health of populations exposed to extreme stressors. 2003. Available at: https://apps.who.int/iris/handle/10665/67866

[11] Pérez DC García AM Carrillo RA Cano JF Cataño SM. Telepsychiatry: a successful experience in Antioquia, Colombia. Rev Colomb Psiquiatr. 2020;49:239–45. 10.1016/j.rcp.2019.06.005 33328016

[12] Schubert NJ Backman PJ Bhatla R Corace KM. Telepsychiatry and patient–provider concordance. Can J Rural Med. 2019;24:75–82. 31249155 10.4103/CJRM.CJRM_9_18

[13] Kruse CS Krowski N Rodriguez B Tran L Vela J Brooks M. Telehealth and patient satisfaction: a systematic review and narrative analysis. BMJ Open. 2017;7:e016242. 10.1136/bmjopen-2017-016242 PMC5629741 28775188

[14] Dham P Gupta N Alexander J Black W Rajji T Skinner E. Community based telepsychiatry service for older adults residing in a rural and remote region-utilization pattern and satisfaction among stakeholders. BMC Psych. 2018;18:1–3. 10.1186/s12888-018-1896-3 PMC6161443 30261845

[15] Hantke N Lajoy M Gould CE. Patient satisfaction with geriatric psychiatry services via video teleconference. Am J Geriatr Psychiatry. 2020;28:491–4. 31530457 10.1016/j.jagp.2019.08.020

[16] Almalky AM Alhaidar FA. Patients’ satisfaction with telepsychiatry services at a University Hospital in Riyadh during the COVID-19 pandemic. Cureus. 2021;13:e17307. 34552838 10.7759/cureus.17307 PMC8449544

